# Soil microbial communities shift along an urban gradient in Berlin, Germany

**DOI:** 10.3389/fmicb.2022.972052

**Published:** 2022-08-12

**Authors:** James Whitehead, Julien Roy, Stefan Hempel, Matthias C. Rillig

**Affiliations:** ^1^Ecology of Plants, Institut für Biologie, Freie Universität Berlin, Berlin, Germany; ^2^Berlin-Brandenburg Institute of Advanced Biodiversity Research (BBIB), Berlin, Germany

**Keywords:** urban soil, fungi, Glomeromycota, bacteria, urban ecology, Berlin, Cercozoa

## Abstract

The microbial communities inhabiting urban soils determine the functioning of these soils, in regards to their ability to cycle nutrients and support plant communities. In an increasingly urbanized world these properties are of the utmost importance, and the microbial communities responsible are worthy of exploration. We used 53 grassland sites spread across Berlin to describe and explain the impacts of urbanity and other environmental parameters upon the diversity and community composition of four microbial groups. These groups were (i) the Fungi, with a separate dataset for (ii) the Glomeromycota, (iii) the Bacteria, and (iv) the protist phylum Cercozoa. We found that urbanity had distinct impacts on fungal richness, which tended to increase. Geographic distance between sites and soil chemistry, in addition to urbanity, drove microbial community composition, with site connectivity being important for Glomeromycotan communities, potentially due to plant host communities. Our findings suggest that many microbial species are well adapted to urban soils, as supported by an increase in diversity being a far more common result of urbanity than the reverse. However, we also found distinctly separate distributions of operational taxonomic unit (OTU)s from the same species, shedding doubt of the reliability of indicator species, and the use of taxonomy to draw conclusion on functionality. Our observational study employed an extensive set of sites across an urbanity gradient, in the region of the German capital, to produce a rich microbial dataset; as such it can serve as a blueprint for other such investigations.

## Introduction

Recent research suggests that humans have heavily impacted the majority of the planet’s ecosystems for at least 12,000 years (Ellis et al., 2021). The pinnacle of human impact occurs within urban landscapes. Definitions of urbanity vary between countries, but in 2014 one estimate of the total land surface covered by urban development was between 2 and 3% (Liu et al., 2014). According to the United Nations, the current trend for urban expansion is likely to result in 68% of people living in urban centers by 2050 (UNDESA Population Division, 2018).

Urban ecosystems contain a unique array of anthropogenic factors, including many relating to global change. For example; summer temperatures in Berlin, Germany, have been reported as reaching ∼10°C higher in highly sealed areas compared to less sealed areas (Dugord, 2013). This situation, known as the urban heat island effect, is expected to become increasingly significant for northern European cities where urban planning has generally not taken high temperatures into account (Ward et al., 2016). Exploring the impacts of these increased temperatures can give advance warning of what to expect from future global temperature spikes. Increased salinity has also been reported in urban landscapes due to irrigation (Ganjegunte et al., 2017) and road de-icing salt, which in some cases reaches levels toxic for land plants (Cunningham et al., 2008). Nitrogen deposition is pervasive in urban ecosystems, although accurately quantifying levels is difficult (Decina et al., 2020). Heavy metal accumulation has also been recorded in many urban ecosystems as a result of anthropogenic activity (e.g., Facchinelli et al., 2001; Lee et al., 2006). However, the impact of heavy metals in urban soils is likely to be buffered: heavy metals are known to demonstrate substantially reduced availability with increasing pH, which has been shown to occur in urban ecosystems due to deposition of alkaline ash and construction dust (Newbound et al., 2010). Urban systems are also likely to experience day-to-day, localized disturbances due to activities such as dog-walking and jogging, the levels of which may determine their impact upon biodiversity. It is possible that anthropogenic activities may even increase biotic diversity according to the intermediate disturbance hypothesis (Connell, 1978).

The relationships between urban systems and biodiversity are still being explored. It appears that different organism groups respond in distinct manners. A general rule of thumb appears to be that in exceptionally urban areas the richness of plants and animals is reduced, whilst at more “standard” urban locations levels of plant richness are increased in comparison to non-urban locations (due in large part to the presence of non-native species), whilst animal richness decreases (McKinney, 2008). Some species of animals (e.g., the house mouse, *Mus musculus*) have been described as living a commensal, or even “anthrodependent,” life with humans which results in a global distribution across urban biomes (Johnson and Munshi-South, 2017). Research in this regard for soil microorganisms is somewhat patchy, most urban soil research having focused on ecosystem services (O’Riordan et al., 2021).

Studies of urban soil microbial diversity have thus far found differing patterns between fungal and bacterial responses to urbanity. Evidence suggests that fungal diversity tends to decrease in urban areas, compared to nearby “natural” locations (Abrego et al., 2020; Tedersoo et al., 2020; Donald et al., 2021), with this pattern even extending to fungal richness being lower in road medians than in urban parks (Reese et al., 2016). Bacterial communities, on the other hand, appear to increase in diversity in urban soils (Delgado-Baquerizo et al., 2021; Donald et al., 2021), with some evidence that protists express the same pattern (Delgado-Baquerizo et al., 2021). However, community composition is also a key component of microbial ecology. Some evidence suggests that globally, urban fungal communities may homogenize and become more similar to one another (Delgado-Baquerizo et al., 2021) with fungal groups favored by urban systems being plant parasites and arbuscular mycorrhizal fungi (AMF) (Donald et al., 2021). Alternatively, urban soils may favor generalists (Abrego et al., 2020). Other patterns observed include a reduced abundance of ectomycorrhizal fungi (Delgado-Baquerizo et al., 2021) and shifts in AMF community composition (Cousins et al., 2003). Bacterial nitrifiers and plant parasites have been reported as showing increased abundance in urban soils (Donald et al., 2021), with a fast-growing lifestyle being favored (Delgado-Baquerizo et al., 2021).

The general list of factors governing microbial biogeographic patterns can be classified into selection, drift, dispersal, and mutation (Vellend, 2010; Hanson et al., 2012). Within urban systems it is as-yet unclear whether selection favors specialists or generalists, and it is also unclear whether dispersal is more heavily constrained by habitat fragmentation, or promoted through transport infrastructure. Highly urban environments may also include niches not seen elsewhere, or the exclusion of normally dominant species due to unfavorable growing conditions. Here, we examined the diversity of soil microorganisms across the urban landscape of Berlin, one of the biggest cities of Europe, with the goal of understanding whether urban soil ecology is shaped by soil chemistry or an intrinsic element of the urban syndrome. Our investigation spanned three kingdoms, namely, Bacteria, Fungi, and Protists. Within the Fungi, we included an additional focus on the plant symbionts, the AMF (phylum Glomeromycota). Within the Protists, we limited our study to the highly diverse phylum Cercozoa (Öztoprak et al., 2020).

Previous research from the same study system as that of this study has observed a change in soil physico-chemical properties in response to an “urban syndrome” of variables (Whitehead et al., 2021). These changes included a reduction in aggregate stability and an increase in water infiltration rate. Both fungi and bacteria influence soil physico-chemical properties through secreting compounds which result in hydrophobicity, thus increasing water run-off potential but also promoting aggregate stability (Mataix-Solera and Doerr, 2004; Epstein et al., 2011). Filamentous fungal hyphae also physically aid in aggregate development and function as translocation routes for unicellular microorganisms (Wick et al., 2007). We decided to investigate Glomeromycotan diversity at a high degree of precision within the fungal kingdom due the pivotal role this group plays in ecosystem functioning, including aggregate formation and stability (Rillig and Mummey, 2006; Leifheit et al., 2014). In addition, AMF colonize 75% of all land plants, playing a vital role in plant fitness (Treseder and Cross, 2006; Brundrett and Tedersoo, 2018). Plants themselves experience varying levels of dispersal limitation and shifts in community assemblage in urban ecosystems depending on their own dispersal traits (Schleicher et al., 2011). It is possible that this may provide a top-down control on Glomeromycotan diversity through host-selection (Grünfeld et al., 2021). The possibility of this is, however, subject to debate (Horn et al., 2017). Previous research, also from the study system used here, has demonstrated an increase in non-mycorrhizal root colonization in response to the urban syndrome, but a resilience of AMF colonization rates (Whitehead et al., 2022). It is as-yet unclear whether there is a similar resilience in Glomeromycotan community composition.

Despite biogeography having been born from the study of protists (Schewiakoff, 1893), it is only in recent years that the importance of protists within soil ecosystems has been truly recognized (Geisen et al., 2018). Containing a degree of diversity previously labeled “near-imponderable” (Foissner, 1999), protists have been described as “puppet-masters” that exhibit top down control on the soil microbiome (Gao et al., 2019). Given the dearth of previous research into urban protist communities and the difficulty entailed in accurately presenting their diversity, we have limited our investigation to the Cercozoa. This highly diverse group of flagellate protists have been demonstrated to influence bacterial communities through selective predation (Glücksman et al., 2010) and are possible to target through the use of a primer pair (Fiore-Donno et al., 2018).

In this study, we examined shifts in soil microbial communities across the urban landscape of Berlin, with the aim of understanding if intrinsically urban variables drive microbial community richness and composition.

## Materials and methods

### Study site

Our study was conducted in Berlin, Germany. The soil textures of Berlin are limited to sand, medium loamy sand and medium silty sand, with a pH range of 4.1–7.5. The yearly average temperature is 9.9°C, with an average yearly rainfall of 976 mm, although recent years have contained periods of reduced rainfall and higher temperatures (von der Lippe et al., 2020). In order to make our results internally comparable we limited our study to dry grasslands. For this study we sampled 53 4 × 4 m grassland plots, all of which were part of the CityScapeLabs research platform (von der Lippe et al., 2020). A wealth of data is available for these sites, discussed below in the section “Database of environmental data.”

Spread across Berlin and into its surrounding federal state, Brandenburg, our study sites represent a gradient of urban locations, consisting of parks, graveyards, forest clearings, road and rail embankments, and derelict land.

### Collection and extraction

The 53 dry grassland plots from the CityScapeLabs research platform used for this study were sampled during the summer of 2017. Fifteen evenly space replicates of 30 cm deep soil cores were taken from each site using a soil-corer and were homogenized in the field in plastic bags, before being divided into three 1 ml Eppendorf tubes. These samples were handled using sterile gloves to prevent contamination, and were temporarily placed in a cooler in the field before being stored in a −20°C freezer. Soil DNA was extracted using the DNeasy PowerSoil Pro Kit (Qiagen, Venlo, Netherlands) following the user instructions.

### Amplification and sequencing

Four taxonomic groups were targeted using specific polymerase chain reaction (PCR) primer pairs. Fungi were identified through the sequencing of the ITS2 region using primers fITS7/ITS4 (Ihrmark et al., 2012). The Glomeromycota were identified through the sequencing of the LSU region using a nested PCR protocol with AMF-specific primers (Krüger et al., 2009) and LR2rev/LR3 (Horn et al., 2014). Bacteria were identified by sequencing the 16S region using primers Eub_338f/Eub_518r (Ghyselinck et al., 2013), and the Protistan division Cercozoa were identified by sequencing the 18S region using primers S963R_Cerco/S947R_Cerco (Fiore-Donno et al., 2018). Following PCR, amplification was checked *via* gel electrophoresis. Samples were purified using solid phase reversible immobilization (SPRI) magnetic beads, indexed, and pooled. DNA concentrations were quantified using first a Qubit and then an Agilent TapeStation. 2*300 bp paired-end Illumina MiSeq sequencing was carried out at the Berlin Center for Genomics in Biodiversity Research (BeGenDiv).

### Bioinformatics and data preparation

Sequencing results were passed through a bioinformatics pipeline in R (R Core Team, 2020) using DADA2 (Callahan et al., 2016), ShortRead (Morgan et al., 2009), and Biostrings (Pagès et al., 2021). The DADA2 pipeline produced 100% similarity operational taxonomic unit (OTUs), however, for a more realistic representation of fungal diversity (Roy et al., 2019; Estensmo et al., 2021), fungal and Glomeromycotan OTUs were reclustered into 97% OTUs. Taxonomic assignments were carried out using the UNITE fungal database for fungal and Glomeromycotan datasets (Nilsson et al., 2019) using the assignTaxonomy() function within DADA2. Glomeromycotan assignments were compared using Blast (Park et al., 2012) to the Krüger et al. (2012) and NCBI databases (NCBI Resource Coordinators, 2016). Bacterial taxonomies were assigned using the Genome Taxonomy Database (Parks et al., 2021) and the Cercozoan taxonomies were assigned using the PR2 database (Guillou et al., 2013). Only assignments with bootstrapped probability values of 100% were kept. Each dataset had some samples where only low numbers of reads were present, these sites were removed leaving the total number of sites represented in each dataset as: Fungi, *n* = 51; Glomeromycota, *n* = 52; Bacteria, *n* = 48; Cercozoa, *n* = 52. The datasets were then normalized using rarefaction to the lowest remaining read count, resulting in read counts in every site of: Fungi, *n* = 30010; Glomeromycota, *n* = 16974; Bacteria, *n* = 20115; Cercozoa, *n* = 10944. This data was used for all of the following analysis, except for the non-metric multidimensional scaling (NMDS), for which Hellinger-transformed data was used.

### Database of environmental data

A database of environmental data was established for these sites in 2017 as part of the CityScapeLabs research platform (von der Lippe et al., 2020). For this study a selection of 45 variables were used, of which 44 were continuous variables and one was categorical, denoting whether sites existed as grasslands prior to 1945, or were only established post-1945. Continuous variables related to site connectivity, site size, slope, plant and litter cover, various measures relating to urbanity, including population density, road density and proximity, railway density and proximity, soil sealing, and distance to the official city center (Flächenschwerpunkt Berlin). Soil chemical properties included N, S, P, K, organic C, and the heavy metals cadmium (Cd), copper (Cu), lead (Pb), Nickel (Ni), and Zinc (Zn), as well as pH, cation exchange capacity and water content. Latitude and longitude were also included. The soil chemical parameters were properties measured in samples taken concurrently to those used in this study. In order to be able to practically use this dataset outliers were removed and the variables were collapsed into 3 main axes of variation *via* principal component analysis (PCA), a technique pioneered in urban ecology by du Toit and Cilliers (2011), for which we used the dudi_pca() function in the ade4 package (Thioulouse et al., 2018). The axes chosen for inclusion in data analysis were selected qualitatively by whether they reflected plausible environmental parameters/syndromes (e.g., urbanity, soil chemistry, see Section “Results”).

### Data analysis

All data analyses were carried out in R (R Core Team, 2020). In order to explore potential drivers of OTU richness, multiple linear regressions were used to examine relationships between OTU richness and each of the three PCA axes, with site age included as an additional predictor. To understand the roles of these variables in shaping community composition, Permutational multivariate analysis of variance (PERMANOVA) of each community data frame was also performed using the same list of predictors, using the adonis() function in vegan (Oksanen et al., 2020).

PERMANOVA models exploring total variance (i.e., where the total variation explained by each predictor, ignoring predictor overlap, was reported) and single predictor-unique variance were performed, both in models including and excluding site age. To visualize community composition, NMDS was performed upon community data using the metaMDS() function in the vegan package with PCA axis scores overlaid using the ordisurf() function. To complement this analysis and explore the relative importance of geographic distance upon community composition, distance-decay analysis was performed using both simple and partial Mantel tests (in the vegan package). To do this, Bray–curtis community distances were correlated with both Euclidean distances between PCA axis scores and geographic distances, which had been calculated using the geosphere package (Hijmans, 2019). Beta diversity partitioning was performed using the “betapart” package (Baselga and Orme, 2012) to reveal the relative importance of species turnover and nestedness.

Heatmaps of the top 20 most abundant OTUs within each dataset were created using the vegan, reshape2 (Wickham, 2007), tidyr (Wickham, 2021), and viridis (Garnier et al., 2021) packages. In order to identify taxonomic groups which showed responses to our environmental axes, we used Kendall correlations to select groups which expressed significant correlations in richness with any of the three PCA axes, using the corrr package (Kuhn et al., 2020). In order to ascertain which environmental parameter was most important in driving the richness of these taxonomic groups, hierarchical partitioning was performed using the package heir.part (Nally and Walsh, 2004). Additionally, indicator species analysis was carried out by splitting the sites into two levels according to their PCA axis 1 score. In order to do this, the 18 sites with the lowest PCA axis 1 scores were categorized as being the most urban. OTUs which were both statistically likely to be indicator species of these highly urban sites, and were assigned at the species level, were recorded. This analysis was carried out using the indicspecies package (De Caceres and Legendre, 2009).

## Results

### Principal component analysis of environmental data

**FIGURE 1:**
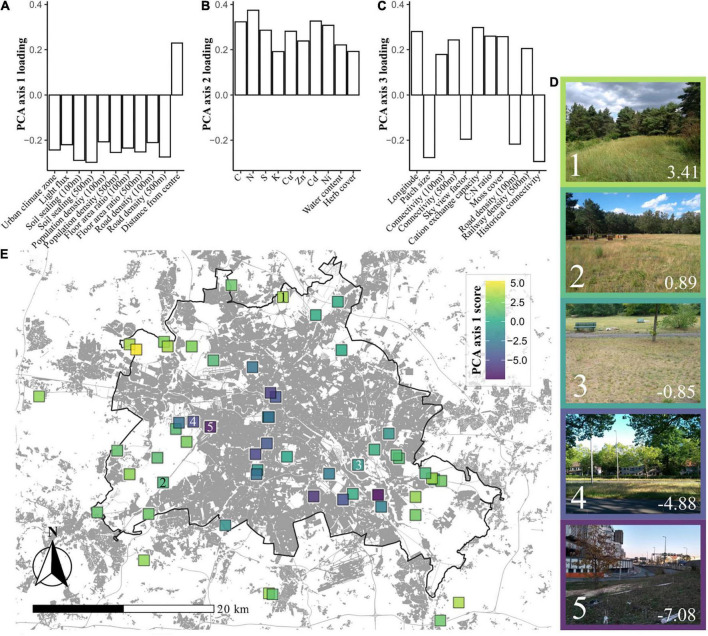
Environmental gradients across grasslands in Berlin. **(A)** Environmental variable loadings within principal component analysis (PCA) axis 1 (21.2% of variance), which can be summarized as being representative of urbanity. **(B)** Variable loadings for PCA axis 2 (10.8%), which can be summarized as being representative of soil chemistry. **(C)** Variable loadings for PCA axis 3 (7.9%), which contains an amalgamation of variables, but with patch size, sky view factor, connectivity, and historical connectivity suggesting that this axis could be seen as something of a vignette of physical site characteristics. **(D)** Examples of sites with differing PCA axis 1 scores, which are denoted in the bottom right hand corner of each image. Numbers in the bottom left hand corners denote locations on the map. **(E)** A map of Berlin with the locations of the sites included in this study. Each site is shaded by its PCA axis 1 score, representing urbanity. Darker coloration indicates more urban sites. The black line represents the border between Berlin and its neighboring federal state, Brandenburg. Areas shaded gray represent built-on land, originally plotted by Anne Hiller (Technisches Universität Berlin), using data from the Landesamt für Umwelt Brandenburg (2009) and Senate Department for Urban Development and Housing (2014).

Using PCA we extracted three axes of environmental variation for the study sites (Figure 1; for biplots of PCA axes see Supplementary Figures 1, 2, and for a list of variable loadings see Supplementary Table 1). PCA axis 1 (21.2% of total variance) reflected a syndrome of urban variables, providing a gradient of “urbanity” across sites, with low scores representing more urban sites. This “urban syndrome” consisted of soil sealing, urban climate zone, floor-area ratio (FAR, a parameter reflecting building development), road density and distance from the city center. PCA axis 2 (10.8% of total variance) reflected a gradient of soil chemical properties including nutrients and heavy metals. Sites with high axis 2 scores generally had high levels of metals such as Cadmium and Zinc, and high levels of Nitrogen. PCA axis 3 (7.9%) reflected a more complex gradient, separating sites according to a collection of variables including some clearly urban-related variables such as site connectivity, and the density of roads and railways. Also included in PCA axis 3 was the size of the grassland patch, and some chemical variables such as C:N ratio and cation exchange capacity.

Principal component analysis axis 1 scores were found to vary between sites established as grasslands before 1945 and those established since (Supplementary Figure 3). This collinearity was taken into account in further statistical analyses.

### Total community composition

A table showing a breakdown of OTU richness for each taxonomic group can be seen in Supplementary Table 2, presented as pie charts in Supplementary Figure 4 and as area plots in Supplementary Figure 5.

The fungal dataset contained 1530510 reads. Reads had a mean length of 286 bases, with a standard deviation of 57. The most abundant phyla present were the Ascomycota (65% of reads), Basidiomycota (23%), Mortierellomycota (6%), Glomeromycota (1%), and the Chytridiomycota (1%). 1% of reads were split between 10 low-abundance phyla (see Supplementary Figure 4A). 3% of reads were unassigned at the phylum level.

The Glomeromycota-specific dataset contained 882648 reads. Reads had a mean length of 382 bases, with a standard deviation of 38. All four orders of the Glomeromycota were present; the Glomerales (54% of reads), the Diversisporales (43%), the Archaeosporales (3%), and Paraglomerales (<1%).

The bacterial dataset contained 965520 reads. The mean read length was 254 bases, with a standard deviation of 14. The most abundant phyla present were the Proteobacteria (18% of reads), Actinobacteriota (14%), Acidobacteriota (14%), Verrucomicrobiota (9%), Bacteroidota (8%), Planctomycetota (5%), and Patescibacteria (1%). 12% of reads were split between 38 low-abundance phyla (see Supplementary Figure 4C). 9% or reads were unassigned at the phylum level.

The Cercozoan dataset contained 569088 reads. The mean read length was 314 bases, with a standard deviation of 14. The most abundant orders present were the Glissomonadida (38% of reads), Cercomonadida (21%), Cryomonadida (15%), Euglyphida (10%), Limnofilida (1%), and Spongomonadida (2%). 5% of reads were split between 14 low-abundance orders (see Supplementary Figure 4D). 7% of reads were unassigned at the order level.

### Drivers of microbial richness

**FIGURE 2:**
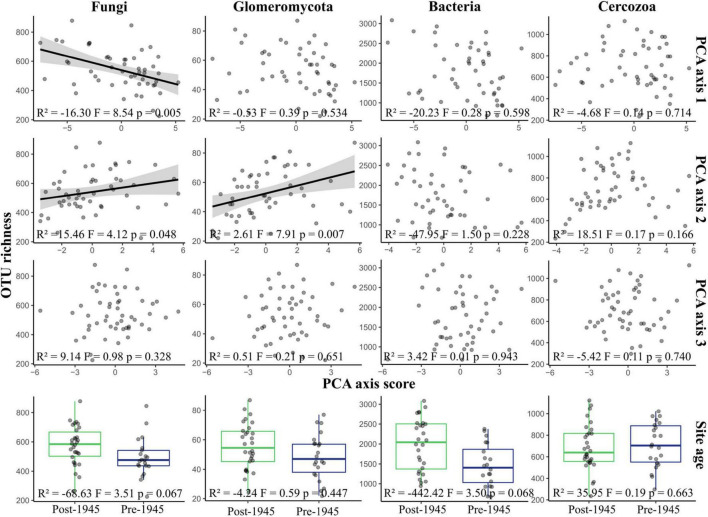
Variation of OTU richness in taxonomic groups across environmental gradients. The figure displays a graphical representation of the results of multiple linear regressions of OTU richness within each taxonomic group. The regression model was Site age + PCA axis 1 + PCA axis 2 + PCA axis 3. Each plot shows richness plotted against site scores for the three PCA axes, representing environmental syndromes, and site age. Please note that lower scores for PCA axis 1 represent more urban sites. Lines are plotted on correlations for which *p* ≤ 0.05; these are linear model regression lines, with gray areas representing 95% confidence areas. Boxplots show the mean, 25th, and 75th percentiles, with whiskers extending to the range, excluding outliers. Statistical results are reported at the bottom of each plot.

There was strong evidence that fungal OTU richness increased with urbanity (PCA axis 1 coefficient = −16.30, *p* = 0.005), and with higher concentrations of nutrients and metal elements (PCA axis 2 coefficient = 15.46, *p* = 0.048), but weak evidence that richness was higher in older sites (coefficient = −68.63, *p* = 0.07) (Figure 2). Glomeromycotan OTU richness was also greater in sites with higher nutrient and heavy metal content (coefficient = 2.61, *p* = 0.007). There was a weak trend for bacterial OTU richness being higher in post-1945 sites than pre-1945 sites (*F* = 3.50, *p* = 0.068), although no relationship was seen with any of the other environmental parameters. Cercozoan OTU richness did not correlate with any of the environmental parameters or site age.

### Drivers of microbial community composition

**FIGURE 3:**
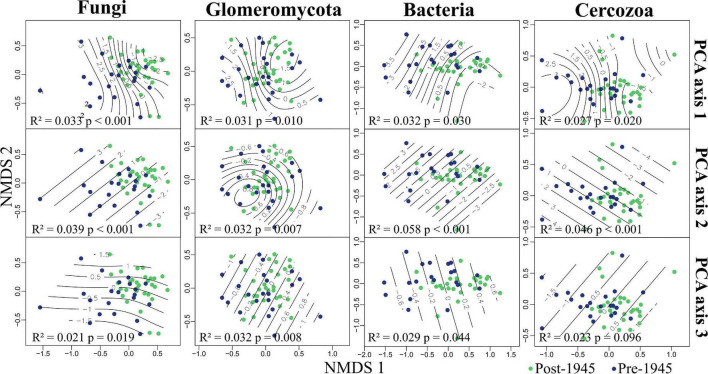
Grassland microbial communities in Berlin, plotted in relation to environmental variation. Non-metric multidimensional scaling (NMDS) plots showing fungal (2D stress = 0.16), Glomeromycotan (2D stress = 0.27), bacterial (2D stress = 0.12), and Cercozoan (2D stress = 0.13) communities segregated by environmental principal component analysis (PCA) axis scores. Reported in each plot are the statistical outputs of marginal PERMANOVA models. Sites are colored by age, the PERMANOVA results for site age are: Fungi, *R*^2^ = 0.021, *p* = 0.216; Glomeromycota, *R*^2^ = 0.020, *p* = 0.345; Bacteria, *R*^2^ = 0.017, *p* = 0.577; Cercozoa, *R*^2^ = 0.019, *p* = 0.341.

PERMANOVA revealed strong evidence for the community composition of all taxonomic groups being driven by both urbanity and soil chemistry (PCA axes 1 and 2; see Figure 3 and Supplementary Table 3). We also found strong evidence for PCA axis 3 (site connectivity and miscellaneous variables) driving Glomeromycotan community composition. There was also weak evidence for the community composition for the other three taxonomic groups being driven by PCA axis 3 (Supplementary Table 3). We found no evidence for the age of sites influencing community composition in any of the taxonomic groups. Represented *via* NMDS, it is clear how communities segregate differently across all three environmental PCA axes, with this pattern being most linear for PCA axes 2 and 3 (Figure 3).

**FIGURE 4:**
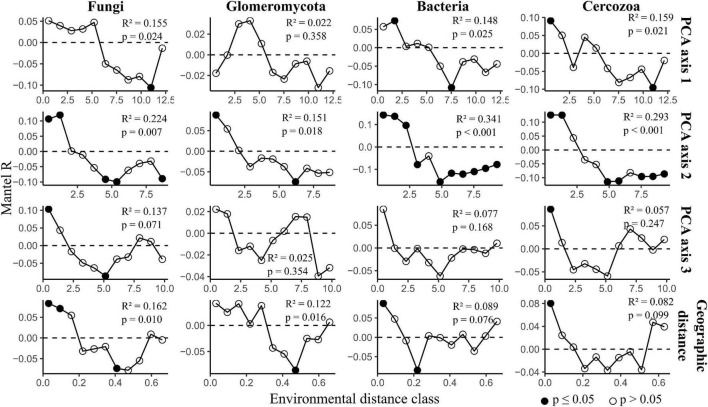
Distance-decay analysis along environmental gradients across grasslands in Berlin. The figure displays mantel correlograms showing community dissimilarity correlated with environmental and geographic distance. Black points indicate strong evidence for differences between distance matrices (*p* ≤ 0.05). The statistical outputs of simple Mantel tests are presented in each plot. The full outputs of simple and partial Mantel tests are reported in Supplementary Table 4.

Distance-decay analysis provided strong evidence for fungal, Glomeromycotan and bacterial community diversity changing with geographic distance, with this trend being particularly strong for the fungi and Glomeromycota (Figure 4). Considering that PCA axis 1 included one geographic parameter (distance from city center) it is notable that for one dataset, the Glomeromycota, PCA axis 1 was not seen to correlate significantly with diversity whilst geographic distance did.

Beta diversity partitioning revealed that for all organism groups, species turnover accounted for the vast majority of beta diversity (Table 1).

**TABLE 1:** Results of beta diversity partitioning for each organism group.

	Fungi	Glomeromycota	Bacteria	Cercozoa
Species turnover	0.952	0.928	0.952	0.942
Nestedness	0.009	0.019	0.013	0.014
Total beta diversity	0.961	0.947	0.964	0.956

Species turnover was measured as Simpson dissimilarity, nestedness as the nestedness-resultant fraction of Sørensen dissimilarity. Total beta diversity was measured as Sørensen dissimilarity.

### Taxonomic breakdown of diversity

The richness of multiple taxonomic groups from each dataset were found to correlate significantly with one or more PCA axes according to Kendall correlations. Using hierarchical partitioning to explore the relative importance of each environmental axis to these taxonomic groups (Table 2), we found evidence that within the fungal dataset, PCA axis 1 was the most significant driver of richness for four phyla, including the Ascomycota (of which it explained 86.3% of variance) and Glomeromycota (85.8%), and the OTUs which were unassigned at the phyla level (80.4%). For all three of these groups, richness was higher at more urban sites (Supplementary Table 5). PCA axis 2 was found to be the most important driver of richness for three fungal phyla, all of which positively correlated with soil nutrient/heavy metal content, including the Mortierellomycota (75.5%). PCA axis 3 was found to be the most important driver of richness for only one phylum, the Basidiomycota (43.6%), which correlated positively with this environmental axis representing site connectivity. This was the only time PCA axis 3 was identified as the most important driver of richness within any of the datasets. Within the Glomeromycotan dataset, PCA axis 1 was found the be the key driver of richness for the Archaeosporales (72.2%), for whom richness was higher in urban sites, and PCA axis 2 was found to be the key driver of richness for the other two groups identified, the Diversisporales (85.7%) and Glomerales (70.9%), the richness of both of which increased with soil nutrient/heavy metal content. Of the nineteen bacterial phyla identified as correlating with an environmental PCA axis, PCA axis 1 was the most significant driver of urbanity for thirteen, with the richness of the remaining six driven by PCA axis 2. Nine Cercozoan orders were identified as correlating with PCA axes, for three of whom PCA axis 1 was the key driver, with the richness of the remaining six orders driven by PCA axis 2. For a complete list of the hierarchical partitioning results of all taxonomic groups please see Supplementary Table 5.

**TABLE 2:** Microbial taxonomic groups for which urbanity [principal component analysis (PCA) axis 1] was identified as the key explanatory variable for shifts in OTU richness.

Fungi (phyla)	Glomeromycota (orders)	Bacteria (phyla)	Cercozoa (orders)
Ascomycota (↑)	Archaeosporales (↑)	Actinobacteriota (↑)	Cercozoa_XX (↑)
Chytridiomycota (↑)		Bacteroidota (↑)	Filosa-Imbricatea_X (↑)
Glomeromycota (↑)		Bdellovibrionota_B (↑)	Plasmodiophorida (↑)
Olpidiomycota (↑)		Chloroflexota_A (↑)	
Unassigned at phyla level (↑)		Eremiobacterota (↓)	
		FCPU426 (↓)	
		Firmicutes_B (↑)	
		Gemmatimonadota (↑)	
		Methylomirabilota (↑)	
		Myxococcota (↑)	
		Nitrospirota (↑)	
		Proteobacteria (↑)	
		Sumerlaeota (↑)	

These groups were identified through hierarchical partitioning of all groups for which Kendall correlations provided strong evidence for environmental variation driving richness. Arrows show whether OTU richness increased (↑) or decreased (↓) in response to increasing urbanity levels (decreasing PCA axis 1 score). Results of hierarchical partitioning for all highlighted taxonomic groups is presented in Supplementary Table 5.

**FIGURE 5:**
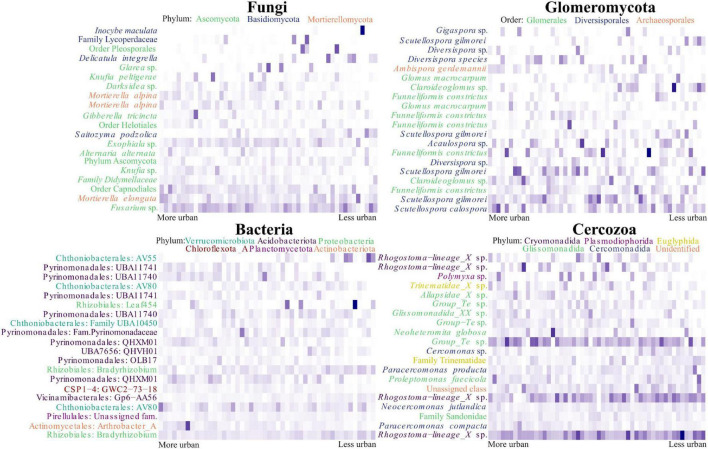
Turnover of the most abundant microbial OTUs across the urbanity gradient in Berlin. The figure displays heatmaps of the top 20 most abundant OTUs within each organism group, arranged across the axis in order of principal component analysis (PCA) axis 1 score. For the fungal and Glomeromycotan OTUs, binomial annotations are included when identified. Otherwise, the highest taxonomic level attributed to the OTU is used. Labels are colored according to phylum in the fungal heatmap and according to order in the Glomeromycotan heatmap. The bacterial heatmap is annotated with the order and genus assigned to OTUs, or family if the genus was unassigned. Labels are colored according to phylum. OTUs in the Cercozoan heatmap are labeled with their binomial name, or the highest taxonomic level assigned. Labels are colored according to order.

One hundred and thirty OTUs within the fungal dataset were identified as being likely urban indicator species (*p* < 0.05) for the eighteen most urban sites (denoted by them having the lowest PCA axis 1 scores), of these OTUs, twenty seven were identified at the species level. We found evidence for *Gibberella tricincta* being a likely indicator species (*p* = 0.047) as well as being in the top 20 most abundant OTUs. However, our heatmap demonstrated an uneven distribution between urban sites, with a peak of abundance in only one site (Figure 5). Also appearing in the list of potential indicator species, and in the top 20 OTUs, were two different OTUs representing *Mortierella alpina* (*p* = 0.017 and 0.029). Within the heatmap these OTUs demonstrated different dispersal between sites and it is likely these OTUs therefore represented separate strains. Within the Glomeromycotan dataset three OTUs were likely indicator species, of which only *Scutellospora calospora* (*p* = 0.032) was identified at species level. This was also the most abundant Glomeromycotan OTU present in the dataset (Figure 5). Within the bacterial dataset 339 OTUs were identified as likely indicator species, of which 127 were identified at the species level. One hundred and three Cercozoan OTUs were identified as likely indicator species, of which eleven were assigned at the species level. *Paracercomonas compacta* (*p* = 0.022) and *Neocercomonas jutlandica* (*p* = 0.047) were both highly abundant OTUs and potential indicator species, despite an apparently relatively wide dispersal (Figure 5). For a list of likely urban indicator species see Supplementary Table 6.

## Discussion

The results of this study indicate that within an urban landscape, soil microbial communities are shaped not only by soil chemistry, but also by factors intrinsically linked to urbanity itself, indeed, total species richness appears to increase with urbanization. Changes in community composition and richness due to urbanization appear to vary between microbial organism groups.

It is important to note that whilst we pick out some individual species for discussion here, these species are selected due to their iconic nature in the literature; the wealth of data available from this study makes it infeasible to comprehensively cover all species.

We found strong evidence for positive relationships between urbanity and the diversity of many microbial phyla, across multiple kingdoms. This pattern was especially clear in the Fungal kingdom, where it can be seen at a higher taxonomic level than in the other organism groups we studied. The response of fungal community richness to urbanity can therefore be compared to that of plant communities, which have also been shown to increase in richness in response to urbanity (McKinney, 2008). Our results contrast with previous observations of fungal richness not changing with urbanity, and bacterial and Cercozoan richness increasing (Delgado-Baquerizo et al., 2021). Other studies have even reported decreases in fungal richness with urbanity (Abrego et al., 2020).

Our study provides evidence that soil microbial communities in urban landscapes are not solely driven by soil chemistry, although this certainly plays a role. Our PCA axis 2, representing soil chemistry, had varying impacts depending on the organism group in question. We found evidence of fungal richness increasing alongside increasing nutrient/heavy metal content. This was also true for the Glomeromycota, in accordance with Tipton et al. (2019), who had previously described the abundance of Glomeromycota in an urban study as being driven by soil chemistry. Despite an absence of this trend in richness in the bacterial and Cercozoan datasets, PERMANOVA and mantel tests both demonstrated strong evidence for soil chemistry shaping all microbial communities. Interestingly, the results of Mantel tests suggested an especially strong impact of soil chemistry on the bacteria and Cercozoa. The small physical size of these organism groups may inhibit their ability to maintain homeostasis in response to a chemical gradient (Luan et al., 2020), and thus explain this trend.

Within the Glomeromycota, we found evidence for urbanity driving an increase in the richness of the Archeosporales (ancestral AMF, sensitive to drought; Canarini et al., 2021), whereas the Diversisporales (thought to be vulnerable to heat or drought, and favor fertile soils; Xiang et al., 2016; Alguacil et al., 2021) and Glomerales (indicators of drought; Canarini et al., 2021) increased in richness in response to increasing soil chemical concentrations.

*Glomus macrocarpum* was present twice within the top 20 most abundant Glomeromycotan OTUs in our study, showing two different patterns of distribution. This was presumably due to two sub-species having differing environmental preferences. It is notable that one subspecies appeared to show highest abundance in the rural and urban areas, whilst the other showed its highest abundance in sites of intermediate urbanity. Interestingly, this species has previously been described as both sensitive (Oehl et al., 2010; Carrenho and Gomes-da-Costa, 2011) and resilient (Sousa et al., 2013) to disturbance. Our observations reaffirm the importance of asking questions about the correct taxonomic resolution for studies such as this (Roy et al., 2019), and provides strong support for van der Heijden et al.’s (2004) suggestion that inferring traits based on taxonomy alone, particularly at the species level, is problematic. In addition, we identified *S. calospora* as a likely indicator species for high urbanity, whilst this species has previously been described as sensitive to disturbance (Gupta et al., 2018) and as a generalist (Oehl et al., 2010).

Another notable way in which our study compares with existing literature is in the case of *Mortierella elongata*. This fungus has previously been identified as a globally abundant indicator species of urban green spaces (Delgado-Baquerizo et al., 2021). Although we did not identify this species as an indicator species of high urbanity, it was the second most abundant OTU within our fungal dataset. It appeared across most sites, with no clear trend relating to urbanity. It should of course be noted that, according to other, less precise, classifications of urbanity used elsewhere, all of our sites could be classed as urban or peri-urban. In addition, all sampling occurred during the summer, and consequently seasonal variation was not taken into account.

One previous study, in the same system as this one, reported an increase in non-mycorrhizal colonization of plant roots in response to urbanity, whilst AMF colonization was not affected (Whitehead et al., 2022). Our observation of an increase in richness of the Ascomycota in response to urbanity, supports the hypothesis that these non-mycorrhizal endophytes were Ascomycota dark septate endophytes. Previous research has suggested that globally, this phylum increases in abundance at urban locations (Delgado-Baquerizo et al., 2021). Whitehead et al. (2022) found that AMF colonization rates in these sites were likely driven by plant community composition, which was in turn driven by a syndrome of parameters very similar to the PCA axis 3 seen in this current study. Given the clear linearity seen in the NMDS plot between Glomeromycotan community, and PCA axis 3, we think it is likely that shifts in the Glomeromycotan community were driven by plant community composition. Indeed, the impacts of plants upon microbial soil communities have been shown to be extensive (Philippot et al., 2013), and it is likely that the shifts in microbial communities we observed in relation to environmental parameters were in some ways mediated by plants.

Our findings of increases in richness of abundant bacterial phyla such as Proteobacteria and Bacteroidota, and less abundant phyla, such as Nitrospira and Gemmatimonadota, in response to urbanity, mimic findings from previous studies (Wang et al., 2018; Delgado-Baquerizo et al., 2021; Stephanou et al., 2021). We also observed urbanity increasing the richness of the Actinobacteria, previously shown to be a significant phylum in cities across China (Xu et al., 2014). The only two taxa for whom we found evidence of a decrease in richness alongside urbanity were the bacterial phyla Eremiobacterota (thought to be acidophilic; Ji et al., 2021) and FCPU426 (potentially associated with cellulose degradation; Doud et al., 2020). It should be noted that for both of these phyla, the relative proportion of variance explained by axis 1 was <50%, suggesting a combination of factors driving this pattern. Urban-related increases in soil pH and decreases in organic matter could explain the reduction in the richness of these phyla.

Cercozoan biogeographical studies are rare in comparison to those of the other groups in this study. However, the lack of similarities between the Cercozoan NMDS plots and those of the other organism groups suggests that within our study the Cercozoa are unlikely to have exhibited top-down control of other soil microbial communities, as previously suggested by Gao et al. (2019). PERMANOVA demonstrated strong evidence for both urbanity and soil chemistry driving Cercozoan community composition with a weak trend for the miscellaneous, but largely connectivity-related, variables of PCA axis 3 also playing a role. Of the three Cercozoan orders we identified as having their richness driven by urbanity, only one, the Plasmodiophorida (a group of plant pathogenic slime molds; Gould, 2009) is well described in terrestrial ecosystems. One OTU from within this order, of the genus Polymyxa (root parasites and vectors for viral parasites, such as Beet Necrotic Yellow Vein Virus; Keskin, 1964), was within our top 20 most abundant Cercozoan OTUs. Whilst the OTU was not clearly distributed across the urbanity gradient, it was, however, most abundant in sites of intermediate and high urbanity. These observations again highlight the role plant diversity is likely to have played in mediating the relationship between urbanity and microbial community assemblage.

The age of sites did not significantly influence the community composition of any of our datasets, despite the collinearity between site age and urbanity. We did, however, observe a weak trend for fungal and bacterial OTU richness decreasing with site age. This finding contrasts those of Hui et al. (2017), who found the reverse in a previous study. This differing result is potentially due to different locations (Berlin vs. Finland) and study sites (varied grasslands vs. lawns in parks). We identified the highest richness of Ascomycota and Nitrospirota in more urban, newer sites, whereas Hui et al. (2017) found these groups to be of highest abundance in older sites.

The impacts of urban landscapes upon organism dispersal are uncertain; both dispersal limitation due to habitat fragmentation and increased dispersal due to roads and railways, or even human movements, could be expected to shape microbial communities. Whilst our study does not investigate this topic in depth, our Mantel correlograms provide evidence for soil microbial communities shifting across the urban landscape, with these changes particularly pronounced in fungal, including Glomeromycotan, communities. These shifts were less pronounced for the bacterial and Cercozoan communities, perhaps due to increased dispersal capacity as a result of their small size (Luan et al., 2020). Within urban landscapes, geographic distance is not a linear measure of habitat dissimilarity and has a non-linear relationship with urban-related variables. This can be seen in the shapes of the Mantel correlograms, whereby sites on opposite sides of the city share more similar communities than to those in the center.

## Conclusion

Within Berlin’s grasslands, urbanity increases the richness of many taxonomic groups across multiple microbial kingdoms, and, alongside other more traditionally recognized environmental features, such as soil chemistry, shapes community composition. Urbanity appears to have a diversifying impact upon fungal communities as a whole, and upon many individual fungal and bacterial phyla, and a few Cercozoan orders. However, the urban Cercozoan community appears to respond less strongly to environmental parameters than other microbial groups. Whilst we did identify individual species that demonstrated particularly strong responses to urbanity, we also found differing responses within the same species, and would therefore urge caution when making functional assessments based on taxonomic findings. Despite this, within the Cercozoa, we noted a high abundance of potentially parasitic species. Whether this is indicative of a general trend deserves further study.

## Data availability statement

Datasets of OTU sequences, abundances, and taxonomic assignments are available at: https://doi.org/10.6084/m9.figshare.20089817. The environmental metadata is available at: https://doi.org/10.6084/m9.figshare.20088632 and https://doi.org/10.6084/m9.figshare.20088707. PCA axis scores for sites are available at: https://doi.org/10.6084/m9.figshare.20088719. Raw sequences are available from the NCBI, accession number PRJNA862455.

## Author contributions

JW, SH, and MR designed the research. JW conducted lab work, bioinformatics, statistics, and wrote first draft of the manuscript. JR created the bioinformatics pipeline and provided guidance on bioinformatics and statistics. All authors added to and edited the text.
