# Supplementary material for: Soil microbial communities shift along an urban gradient in Berlin, Germany
Source: Front Microbiol. 2022 Aug 12;13:972052. doi: 10.3389/fmicb.2022.972052 (PMC9412169; doi:10.3389/fmicb.2022.972052)
Supplement: Supplementary file 1 [file Data_sheet_1.docx]

# **Supplementary materials**


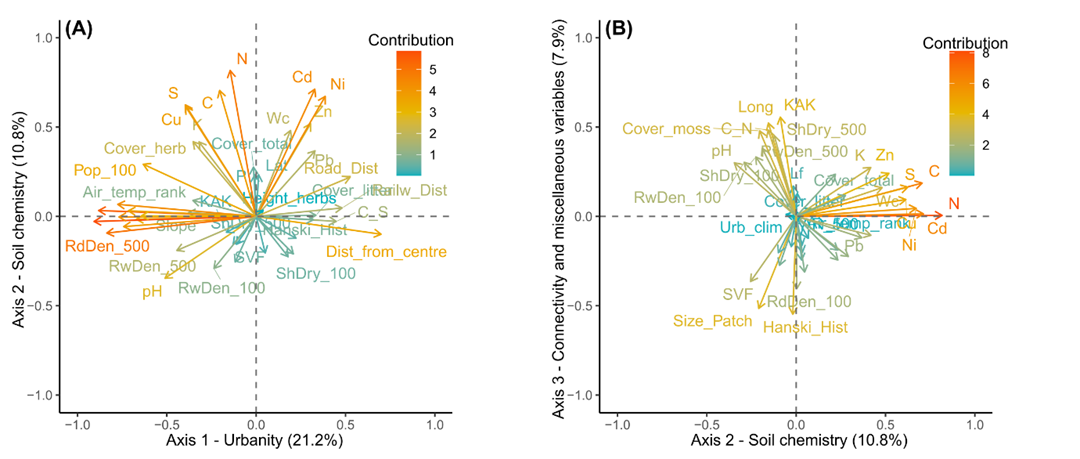


**Supplementary figure 1**. Biplots of the PCA of environmental variables. (A) PCA axes 1 and 2. (B) PCA axes 2 and 3. Percentages of total variance are presented by axis labels. Abbreviations for variables are presented in Supplementary table 1.


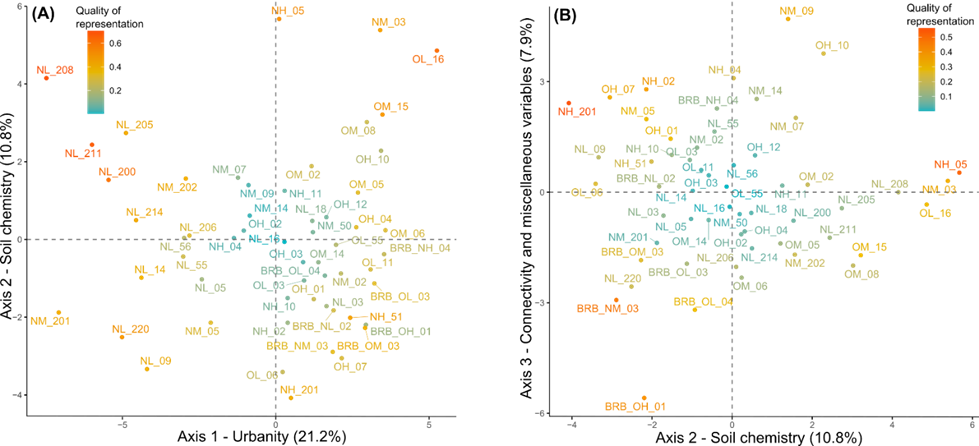


**Supplementary figure 2.** Plots of study sites according to their PCA scores. (A) Axes 1 and 2. (B) Axes 2 and 3.

**
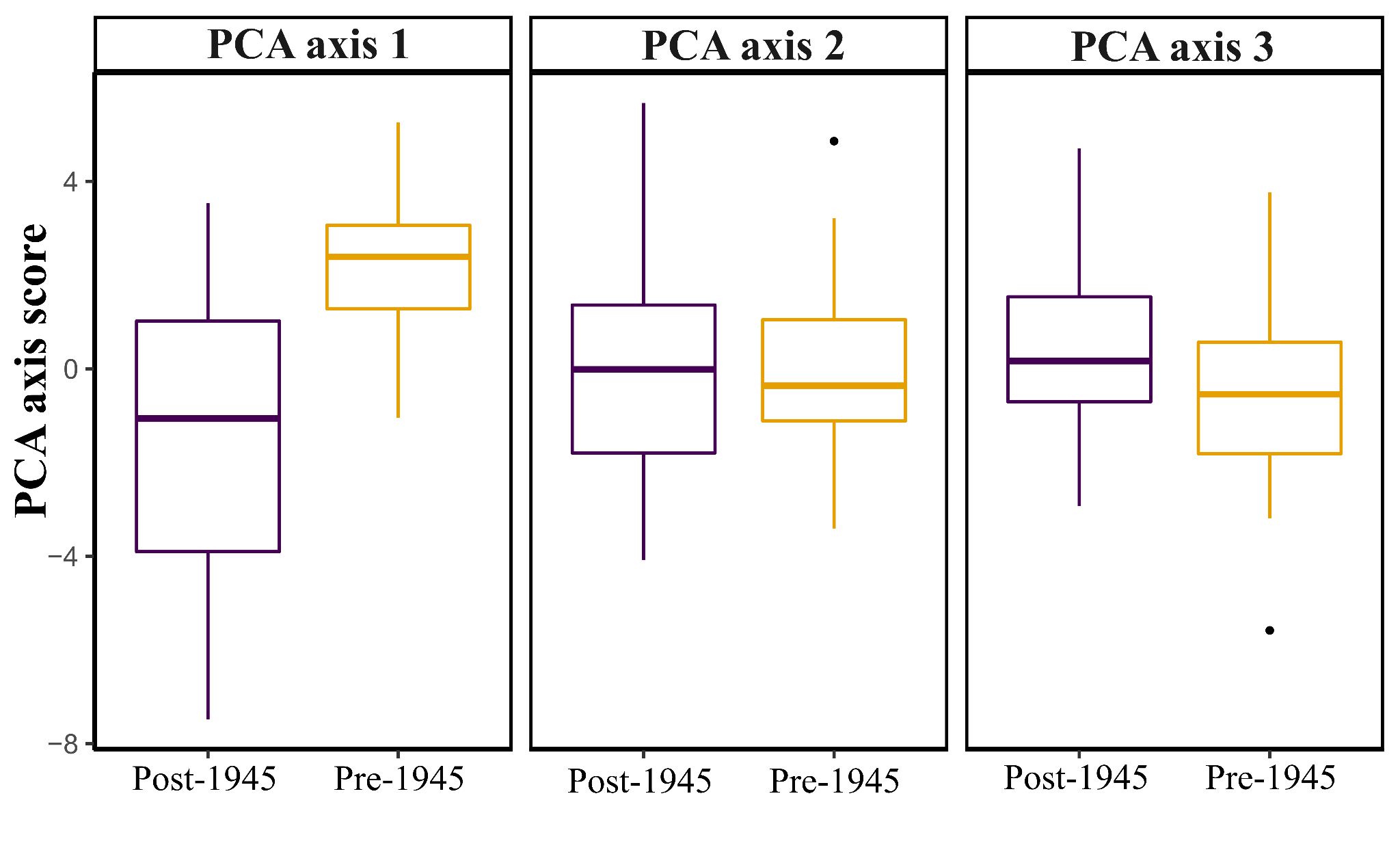
**

**Supplementary figure 3.** Boxplot of PCA scores, separated by different age-classes of site. Please note, higher PCA axis 1 score means sites are less urban than those with lower scores.

**Supplementary table 1.** PCA variable loadings for environmental data. These variables are plotted in biplots in Supplementary Figure 1.

| **Variable abbreviation** | **Environmental variable** | **Axis 1** | **Axis 2** | **Axis 3** |
| --- | --- | --- | --- | --- |
| Long | Longitude | -0.04227 | -0.07136 | 0.280696 |
| Lat | Latitude | 0.004518 | 0.10704 | -0.13103 |
| Size_Patch | Patch size | 0.068194 | -0.09608 | -0.27659 |
| ShDry_100 | Patch connectivity (100m) | 0.058653 | -0.10354 | 0.178869 |
| ShDry_500 | Patch connectivity (500m) | 0.073249 | -0.05616 | 0.242745 |
| Air_temp_rank | Air temperature | -0.11615 | 0.040923 | 0.023029 |
| Urb_clim | Urban climate zone | -0.24311 | -0.02614 | 0.006044 |
| SVF | Sky view factor | -0.03849 | -0.1171 | -0.19566 |
| pH | pH | -0.16724 | -0.15885 | 0.161177 |
| C | Organic C | -0.06703 | 0.323445 | 0.100279 |
| N | Nitrogen | -0.0477 | 0.374607 | 0.003028 |
| S | Sulphur | -0.13002 | 0.28663 | 0.088025 |
| P | Phosphorous | -0.00251 | 0.107148 | -0.10118 |
| K | Potassium | -0.10492 | 0.191407 | 0.146732 |
| Cu | Copper | -0.1295 | 0.281841 | 0.050615 |
| Zn | Zinc | 0.099866 | 0.238147 | 0.129596 |
| Cd | Cadmium | 0.107571 | 0.326857 | 0.007538 |
| Pb | Lead | 0.107995 | 0.168502 | -0.06402 |
| Ni | Nickel | 0.126567 | 0.308139 | 0.024159 |
| Wc | Water content | 0.062939 | 0.220806 | 0.086501 |
| Lf | Electron flux | -0.21989 | -0.00736 | 0.091332 |
| KAK | Cation exchange capacity | -0.0546 | -0.04074 | 0.298374 |
| C_N | C:N ratio | -0.03626 | -0.07164 | 0.259529 |
| C_S | C:S ratio | 0.146928 | -0.01278 | -0.09077 |
| Slope | Slope | -0.12443 | 0.009522 | -0.06163 |
| Cover_total | Total plant cover | -0.00499 | 0.126734 | 0.063009 |
| Cover_herb | Herbaceous plant cover | -0.11573 | 0.191926 | -0.05669 |
| Cover_moss | Moss cover | 0.016616 | -0.09397 | 0.257433 |
| Cover_litter | Plant litter cover | 0.109648 | 0.003004 | 0.069054 |
| Height_herbs | Height of herbaceous plants | 0.012718 | 0.01591 | -0.10854 |
| Seal_100 | Soil sealing (100m) | -0.28898 | 0.015073 | -0.08716 |
| Seal_500 | Soil sealing (500m) | -0.29745 | -0.01293 | -0.00033 |
| Pop_100 | Population (100m) | -0.20718 | 0.134006 | -0.12049 |
| Pop_500 | Population (500m) | -0.25398 | 0.03096 | -0.06599 |
| FAR_100 | Floor area ratio (100m) | -0.23459 | 0.014356 | -0.1314 |
| FAR_500 | Floor area ratio (500m) | -0.25175 | -0.00145 | -0.01996 |
| RdDen_100 | Road density (100m) | -0.21052 | 0.001619 | -0.21729 |
| RdDen_500 | Road density (500m) | -0.27406 | -0.04277 | -0.14694 |
| Road_Dist | Distance to nearest road | 0.172214 | 0.100789 | 0.126739 |
| RwDen_100 | Railway density (100m) | -0.07737 | -0.13363 | 0.162583 |
| RwDen_500 | Railway density (500m) | -0.14554 | -0.08738 | 0.205132 |
| Railw_Dist | Distance to nearest railway | 0.157683 | 0.021756 | -0.1674 |
| Dist_from_centre | Distance from city centre | 0.229113 | -0.04579 | -0.10906 |
| Hanski_Hist | Historical site connectivity | 0.104988 | -0.00865 | -0.294 |

**Supplementary table 2.** A breakdown of OTU richness and read numbers which are present in each organism dataset used in this study.

| **Dataset (total OTUs and reads)** | **Taxonomic group** | **OTUs (percentage of total)** | **Reads (percentage of total)** |
| --- | --- | --- | --- |
| **Fungi**  6805 OTUs  1530510 reads | Ascomycota | 2658 (39%) | 993032 (65%) |
|  | Basidiomycota | 1356 (20%) | 354757 (23%) |
|  | Glomeromycota | 369 (5%) | 16079 (1%) |
|  | Chytridiomoycota | 131 (2%) | 14561 (1%) |
|  | Mortierellomycota, | 119 (2%) | 90116 (6%) |
|  | Unassigned at phylum level | 1995 (29%) | 44912 (3%) |
|  | 10 other phyla: see Supp. Fig. 4 | 177 (3%) | 17053 (1%) |
| **Glomeromycota**  318 OTUs  882648 reads | Glomerales | 209 (66%) | 478423 (54%) |
|  | Diversisporales | 89 (28%) | 377819 (43%) |
|  | Archaesporales | 16 (5%) | 26073 (3%) |
|  | Paraglomerales | 4 (1%) | 333 (<1%) |
| **Bacteria**  27469 OTUs  965520 reads | Proteobacteria | 3611 (13%) | 172934 (18%) |
|  | Planctomycetota | 3198 (12%) | 52110 (5%) |
|  | Bacteroidota | 2805 (10%) | 81169 (8%) |
|  | Actinobacteriota, | 2493 (9%) | 139341 (14%) |
|  | Acidobacteriota | 2486 (9%) | 215623 (14%) |
|  | Patescibacteria | 2256 (8%) | 14362 (1%) |
|  | Verrucomicrobiota | 1515 (6%) | 87091 (9%) |
|  | Unassigned at phylum level | 4905 (18%) | 82836 (9%) |
|  | 38 other phyla: see Supp. Fig. 4 | 4200 (15%) | 120054 (12%) |
| **Cercozoa**  7330 OTUs  569088 reads | Glissomonadida | 2916 (40%) | 217554 (38%) |
|  | Cercomonadida | 1248 (17%) | 120220 (21%) |
|  | Cryomonadida | 954 (13%) | 84382 (15%) |
|  | Euglyphida | 447 (6%) | 56482 (10%) |
|  | Limnofilida | 194 (3%) | 7253 (1%) |
|  | Spongomonadida | 123 (2%) | 11652 (2%) |
|  | Unassigned at the order level | 927 (13%) | 40612 (7%) |
|  | 14 other orders: see Supp. Fig. 4 | 521 (7%) | 30960 (5%) |

**
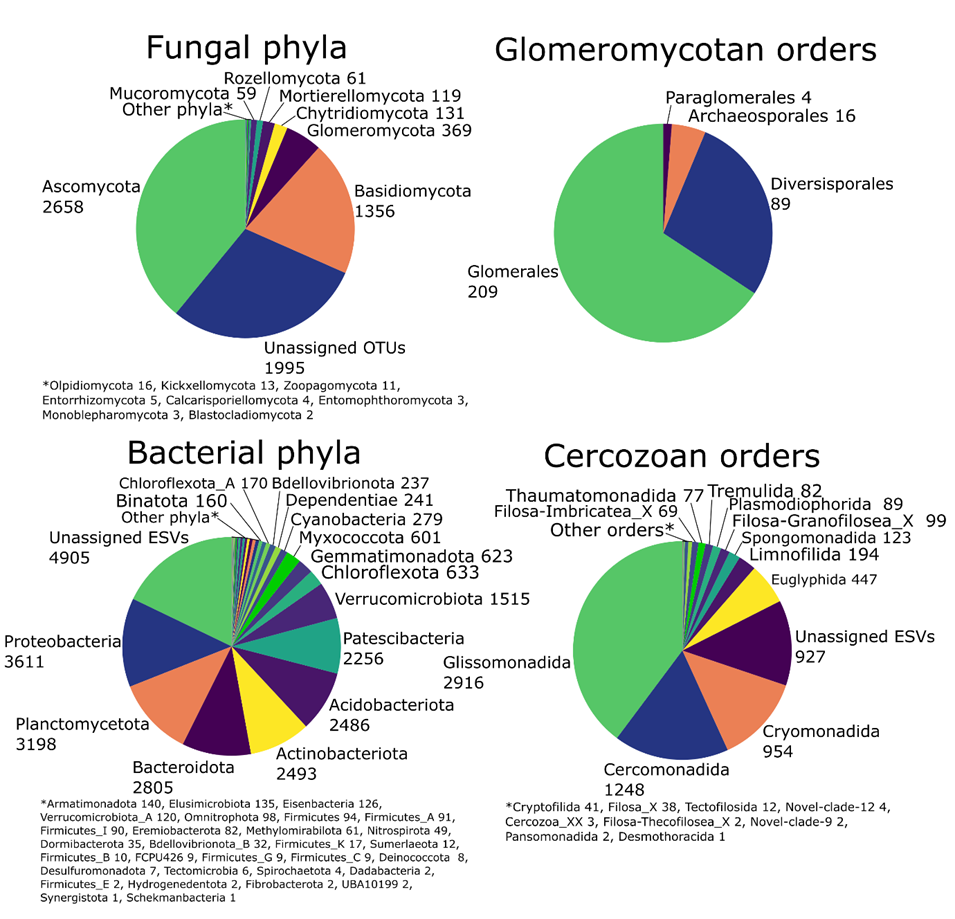
**

**Supplementary Figure 4.** Pie charts of the relative numbers of OTUs present in each of the four datasets used in this study. The taxonomic levels at which this is presented are the following: Fungi, phylum level; Glomeromycota, order level; Bacteria, phylum level; Cercozoa, order level.


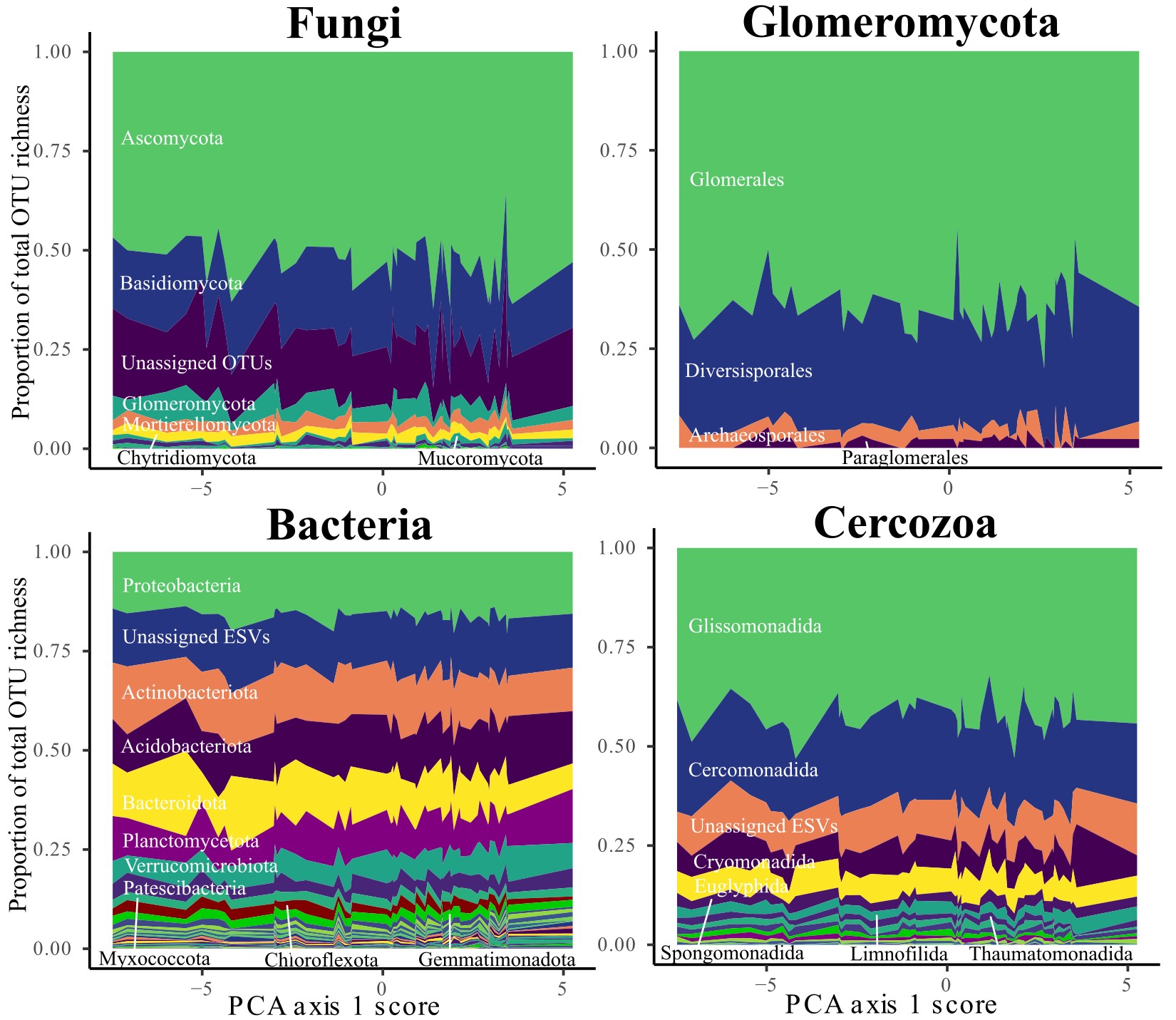


**Supplementary Figure 5.** Area plots of relative proportions of reads attributed to different taxonomic groups within each dataset across the urbanity gradient. For the fungi, the low-read non-labelled phyla are: Rozellomycota, Olpidiomycota, Zoopagomycota, Kickxellomycota, Calcarisporiellomycota, Entorrhizomycota, Monoblepharomycota, Entomophthoromycota, Blastocladiomycota.

Low-read bacterial phyla present but not labelled in the figure are: Chloroflexota_A, Binatota, Cyanobacteria, Firmicutes, Methylomirabilota, Nitrospirota, Bdellovibrionota, Firmicutes_I, Dependentiae, Armatimonadota, Eisenbacteria, Elusimicrobiota, Firmicutes_A, Eremiobacterota, Omnitrophota, Verrucomicrobiota_A, Dormibacterota, Bdellovibrionota_B, Firmicutes_K, Sumerlaeota, Deinococcota, Desulfuromonadota, FCPU426, Firmicutes_B, Firmicutes_G, Firmicutes_C, Dadabacteria, Spirochaetota, Tectomicrobia, Firmicutes_E, Hydrogenedentota, Fibrobacterota, UBA10199, Synergistota and Schekmanbacteria.

Low-read cercozoan orders present but not labelled are: Filosa-Imbricatea_X, Filosa-Granofilosea_X, Tremulida, Plasmodiophorida, Filosa_X, Cryptofilida, Tectofilosida, Cercozoa_XX, Filosa-Thecofilosea_X, Novel-clade_12, Desmothoracida.

**Supplementary table 3.** PERMANOVA output, using both sequential and partial models, 9999 permutations

|  |  |  | **Fungi** | | | **Glomeromycota** | | | **Bacteria** | | | **Cercozoa** | | |
| --- | --- | --- | --- | --- | --- | --- | --- | --- | --- | --- | --- | --- | --- | --- |
|  |  |  | **R2** | **F** | **p** | **R2** | **F** | **p** | **R2** | **F** | **p** | **R2** | **F** | **p** |
| **Sequential model** | **Axes1, 2 and 3** | **Axis 1** | 0.048 | 2.570 | <0.001 | 0.028 | 1.483 | 0.033 | 0.065 | 3.366 | <0.001 | 0.051 | 2.808 | <0.001 |
|  |  | **Axis 2** | 0.039 | 2.061 | <0.001 | 0.032 | 1.710 | 0.006 | 0.057 | 2.982 | <0.001 | 0.046 | 2.496 | <0.001 |
|  |  | **Axis 3** | 0.026 | 1.382 | 0.0148 | 0.030 | 1.607 | 0.011 | 0.032 | 1.650 | 0.030 | 0.025 | 1.343 | 0.053 |
|  | **Axes 1, 2, 3 and age** | **Axis 1** | 0.048 | 2.575 | <0.001 | 0.028 | 1.485 | 0.028 | 0.065 | 3.358 | <0.001 | 0.051 | 2.810 | <0.001 |
|  |  | **Axis 2** | 0.679 | 2.065 | 0.012 | 0.032 | 1.713 | 0.006 | 0.057 | 2.975 | <0.001 | 0.046 | 2.498 | <0.001 |
|  |  | **Axis 3** | 0.026 | 1.386 | 0.012 | 0.030 | 1.609 | 0.013 | 0.032 | 1.646 | 0.031 | 0.025 | 1.344 | 0.051 |
|  |  | **Age** | 0.021 | 1.090 | 0.210 | 0.020 | 1.070 | 0.337 | 0.017 | 0.898 | 0.585 | 0.019 | 1.031 | 0.347 |
| **Marginal model** | **Axes 1, 2 and 3** | **Axis 1** | 0.048 | 2.556 | <0.001 | 0.027 | 1.451 | 0.039 | 0.057 | 2.957 | <0.001 | 0.050 | 2.749 | <0.001 |
|  |  | **Axis 2** | 0.039 | 2.055 | <0.001 | 0.033 | 1.719 | 0.004 | 0.058 | 3.009 | <0.001 | 0.046 | 2.500 | <0.001 |
|  |  | **Axis 3** | 0.026 | 1.383 | 0.011 | 0.030 | 1.607 | 0.014 | 0.032 | 1.650 | 0.029 | 0.025 | 1.343 | 0.051 |
|  | **Axes 1, 2, 3 and age** | **Axis 1** | 0.033 | 1.732 | <0.001 | 0.031 | 1.633 | 0.010 | 0.032 | 1.660 | 0.030 | 0.027 | 1.500 | 0.020 |
|  |  | **Axis 2** | 0.039 | 2.069 | <0.001 | 0.032 | 1.699 | 0.007 | 0.058 | 3.012 | <0.001 | 0.046 | 2.513 | <0.001 |
|  |  | **Axis 3** | 0.021 | 1.344 | 0.019 | 0.032 | 1.666 | 0.008 | 0.029 | 1.521 | 0.044 | 0.023 | 1.238 | 0.096 |
|  |  | **Age** | 0.021 | 1.090 | 0.216 | 0.020 | 1.070 | 0.345 | 0.017 | 0.898 | 0.577 | 0.019 | 1.031 | 0.341 |

**Supplementary Table 4.** Results of simple and partial mantel tests, using Pearson correlations and 9999 permutations.

|  |  | **Fungi** | | **Glomeromycota** | | **Bacteria** | | **Cercozoa** | |
| --- | --- | --- | --- | --- | --- | --- | --- | --- | --- |
|  | **Model** | **R2** | **p** | **R2** | **p** | **R2** | **p** | **R2** | **p** |
| Simple Mantel tests | PCA axis 1 | 0.155 | 0.024 | 0.022 | 0.358 | 0.148 | 0.025 | 0.159 | 0.021 |
|  | PCA axis 2 | 0.224 | 0.007 | 0.151 | 0.018 | 0.341 | <0.001 | 0.293 | <0.001 |
|  | PCA axis 3 | 0.137 | 0.071 | 0.025 | 0.354 | 0.077 | 0.168 | 0.057 | 0.247 |
|  | Geographic distance | 0.162 | 0.010 | 0.122 | 0.016 | 0.089 | 0.076 | 0.082 | 0.099 |
| Partial Mantel tests | Axis 1 + geographic distance | 0.164 | 0.023 | 0.028 | 0.315 | 0.154 | 0.023 | 0.162 | 0.021 |
|  | Axis 2 + geographic distance | 0.225 | 0.006 | 0.152 | 0.017 | 0.342 | <0.001 | 0.294 | <0.001 |
|  | Axis 3 + geographic distance | 0.110 | 0.114 | 0.003 | 0.477 | 0.063 | 0.201 | 0.040 | 0.309 |
|  | Axis 1 + Axis 2 | 0.133 | 0.042 | 0.004 | 0.469 | 0.124 | 0.046 | 0.136 | 0.040 |
|  | Axis 1 + Axis 3 | 0.163 | 0.022 | 0.023 | 0.349 | 0.153 | 0.021 | 0.163 | 0.019 |
|  | Axis 2 + Axis 3 | 0.234 | 0.004 | 0.153 | 0.015 | 0.346 | <0.001 | 0.296 | <0.001 |

**Supplementary table 5.** Results of hierarchical partitioning of taxonomic group richness. Presented are the most important PCA axis alongside the percentage of variance this represents within each taxonomic group, and the Kendall correlation coefficient between this axis and the OTU richness of the taxonomic group.

| **Dataset** | **Taxonomic group** | **Most significant**  **axis** | **Explained**  **variance** | **Correlation**  **coefficient** |
| --- | --- | --- | --- | --- |
| **Fungi** | Ascomycota | Axis 1 | 86.3% | -0.30 |
|  | Basidiomycota | Axis 3 | 43.6% | 0.22 |
|  | Chytridiomycota | Axis 1 | 68.4% | -0.26 |
|  | Glomeromycota | Axis 1 | 85.8% | -0.33 |
|  | Mortierellomycota | Axis 2 | 75.5% | 0.31 |
|  | Olpidiomycota | Axis 1 | 100.0% | -0.45 |
|  | Rozellomycota | Axis 2 | 84.4% | 0.32 |
|  | Zoopagomycota | Axis 2 | 67.8% | 0.24 |
|  | OTUs unassigned at phyla level | Axis 1 | 80.4% | -0.32 |
| **Glomeromycota** | Archaeosporales | Axis 1 | 72.2% | -0.23 |
|  | Diversisporales | Axis 2 | 85.7% | 0.22 |
|  | Glomerales | Axis 2 | 70.9% | 0.28 |
| **Bacteria** | Actinobacteriota | Axis 1 | 81.2% | -0.32 |
|  | Armatimonadota | Axis 2 | 87.6% | -0.20 |
|  | Bacteroidota | Axis 1 | 69.9% | -0.30 |
|  | Bdellovibrionota_B | Axis 1 | 87.8% | -0.27 |
|  | Chloroflexota | Axis 2 | 70.4% | -0.43 |
|  | Chloroflexota_A | Axis 1 | 87.8% | -0.38 |
|  | Deinococcota | Axis 2 | 82.8% | -0.39 |
|  | Dormibacterota | Axis 2 | 50.2% | 0.33 |
|  | Eremiobacterota | Axis 1 | 48.1% | 0.29 |
|  | FCPU426 | Axis 1 | 43.6% | 0.27 |
|  | Firmicutes_B | Axis 1 | 95.4% | -0.31 |
|  | Gemmatimonadota | Axis 1 | 65.3% | -0.24 |
|  | Methylomirabilota | Axis 1 | 49.1% | -0.28 |
|  | Myxococcota | Axis 1 | 51.6% | -0.22 |
|  | Nitrospirota | Axis 1 | 69.9% | -0.26 |
|  | Proteobacteria | Axis 1 | 53.4% | -0.21 |
|  | Sumerlaeota | Axis 1 | 70.0% | -0.25 |
|  | UBA10199 | Axis 2 | 50.0% | -0.24 |
|  | OTUs unassigned at phyla level | Axis 2 | 56.6% | -0.20 |
| **Cercozoa** | Cercomonadida | Axis 2 | 98.4% | 0.23 |
|  | Cercozoa_XX | Axis 1 | 68.2% | -0.37 |
|  | Cryptofilida | Axis 2 | 51.8% | 0.24 |
|  | Filosa_X | Axis 2 | 96.5% | 0.34 |
|  | Filosa-Imbricatea_X | Axis 1 | 64.4% | -0.25 |
|  | Filosa-Thicofilosea_X | Axis 2 | 89.0% | 0.21 |
|  | Limnofilida | Axis 2 | 85.5% | 0.40 |
|  | Plasmodiophorida | Axis 1 | 97.8% | -0.32 |
|  | Tremulida | Axis 2 | 73.1% | 0.24 |

**Supplementary table 6.** Species which are likely to be indicators of highly urban sites. Presented are the results of indicator species analysis for the top 18 most urban sites according to PCA axis 1 score.

| **Dataset** | **Species** | **Point biserial correlation coefficient** | **p value** |
| --- | --- | --- | --- |
| **Fungi** | *Septoglomus viscosum* | 0.446 | 0.0026 |
|  | *Purpureocillium lilacinum* | 0.437 | 0.0032 |
|  | *Ganoderma adspersum* | 0.403 | 0.0091 |
|  | *Keissleriella culmifida* | 0.378 | 0.0318 |
|  | *Claroideoglomus claroideum* | 0.378 | 0.0051 |
|  | *Clonostachys rosea* | 0.375 | 0.0186 |
|  | *Thanatephorus cucumeris* | 0.375 | 0.004 |
|  | *Mortierella alpina* | 0.371 | 0.0165 |
|  | *Olpidium brassicae* | 0.365 | 0.0079 |
|  | *Septoglomus viscosum* | 0.363 | 0.0137 |
|  | *Thanatephorus cucumeris* | 0.362 | 0.019 |
|  | *Robillarda sessilis* | 0.358 | 0.034 |
|  | *Holtermanniella takashimae* | 0.343 | 0.0171 |
|  | *Glomus aggregatum* | 0.341 | 0.0191 |
|  | *Nectria ramulariae* | 0.339 | 0.0269 |
|  | *Buckleyzyma aurantiaca* | 0.339 | 0.0205 |
|  | *Schizothecium conicum* | 0.333 | 0.0096 |
|  | *Mortierella antarctica* | 0.329 | 0.0098 |
|  | *Preussia flanaganii* | 0.317 | 0.0053 |
|  | *Tolypocladium album* | 0.309 | 0.0499 |
|  | *Myrmecridium phragmitis* | 0.305 | 0.0054 |
|  | *Lectera longa* | 0.304 | 0.032 |
|  | *Mucor hiemalis* | 0.301 | 0.0302 |
|  | *Filobasidium stepposum* | 0.296 | 0.0094 |
|  | *Gibberella tricincta* | 0.282 | 0.0466 |
|  | *Naganishia adeliensis* | 0.273 | 0.0165 |
|  | *Mortierella alpina* | 0.236 | 0.029 |
| **Glomeromycota** | *Scutellospora calospora* | 0.284 | 0.0319 |
| **Bacteria** | *Nitrospira C japonica*(RS_GCF_900169565.1) | 0.556 | 4.00E-04 |
|  | SCN-69-37_sp001724025(GB_GCA_001724025.1) | 0.538 | 1.00E-04 |
|  | QHWT01_sp003222675(GB_GCA_003222675.1) | 0.528 | 4.00E-04 |
|  | UBA6082_sp002428665(GB_GCA_002428665.1) | 0.52 | 0.0024 |
|  | *Solirubrobacter soli*(RS_GCF_000423665.1) | 0.519 | 9.00E-04 |
|  | Gp1-AA17_sp003223515(GB_GCA_003223515.1) | 0.512 | 9.00E-04 |
|  | AV55_sp003219415(GB_GCA_003219415.1) | 0.51 | 3.00E-04 |
|  | *Lysobacter*_sp001427225(RS_GCF_001427225.1) | 0.506 | 7.00E-04 |
|  | SCN-70-22_sp001724275(GB_GCA_001724275.1) | 0.498 | 0.0012 |
|  | QHWT01_sp003222675(GB_GCA_003222675.1) | 0.497 | 6.00E-04 |
|  | OLB17_sp001567505(GB_GCA_001567505.1) | 0.479 | 0.0014 |
|  | *Microlunatus phosphovorus*(RS_GCF_000270245.1) | 0.469 | 0.0012 |
|  | *Cellulomonas*_sp000426185(RS_GCF_000426185.1) | 0.465 | 0.0025 |
|  | *Luteitalea pratensis*(RS_GCF_001618865.1) | 0.457 | 0.0028 |
|  | *Pedobacter panaciterrae*(RS_GCF_001636695.1) | 0.455 | 0.0033 |
|  | UBA11741_sp002427845(GB_GCA_002427845.1) | 0.453 | 0.0034 |
|  | *Williamsia*_sp002095395(RS_GCF_002095395.1) | 0.453 | 0.0021 |
|  | *Oligoflexus tunisiensis*(GB_GCA_001748245.1) | 0.449 | 0.0043 |
|  | *Fimbriiglobus ruber*(RS_GCF_002197845.1) | 0.446 | 0.0039 |
|  | *Solirubrobacter soli*(RS_GCF_000423665.1) | 0.443 | 0.0047 |
|  | *Nonomuraea solani*(RS_GCF_900108335.1) | 0.442 | 0.0068 |
|  | *Haloferula*_sp000739615(RS_GCF_000739615.1) | 0.441 | 0.0025 |
|  | HRBIN40_sp002898275(GB_GCA_002898275.1) | 0.441 | 0.0055 |
|  | *Chitinophaga*_sp900110995(RS_GCF_900110995.1) | 0.439 | 0.0058 |
|  | *Actinoplanes atraurantiacus*(RS_GCF_900215205.1) | 0.435 | 0.0036 |
|  | UBA11740_sp003168335(GB_GCA_003168335.1) | 0.433 | 0.0039 |
|  | *Luteitalea pratensis*(RS_GCF_001618865.1) | 0.429 | 0.0134 |
|  | *Litorilinea aerophila*(GB_GCA_002148365.1) | 0.428 | 0.0018 |
|  | *Luteitalea pratensis*(RS_GCF_001618865.1) | 0.425 | 0.0074 |
|  | GWC2-73-18_sp001794945(GB_GCA_001794945.1) | 0.424 | 0.005 |
|  | *Ohtaekwangia koreensis*(RS_GCF_900167975.1) | 0.423 | 0.0072 |
|  | *Fimbriiglobus ruber*(RS_GCF_002197845.1) | 0.423 | 0.0091 |
|  | *Agromyces*_sp001429165(RS_GCF_001429165.1) | 0.418 | 0.0038 |
|  | UNC496MF_sp900116125(RS_GCF_900116125.1) | 0.418 | 0.0263 |
|  | UBA11740_sp003168335(GB_GCA_003168335.1) | 0.417 | 0.0275 |
|  | *Aquamicrobium*_sp001427385(RS_GCF_001427385.1) | 0.415 | 0.0274 |
|  | RBG-16-40-8_sp001769925(GB_GCA_001769925.1) | 0.411 | 0.0148 |
|  | Gp7-AA10_sp003223695(GB_GCA_003223695.1) | 0.41 | 0.006 |
|  | UBA2421_sp002343075(GB_GCA_002343075.1) | 0.408 | 0.0162 |
|  | *Nocardioides*_sp001425025(RS_GCF_001425025.1) | 0.408 | 0.006 |
|  | *Chthoniobacter flavus*(RS_GCF_000173075.1) | 0.408 | 0.0104 |
|  | *Arthrobacter*_A_sp003268655(GB_GCA_003268655.1) | 0.408 | 0.0022 |
|  | Gp6-AA56_sp003222395(GB_GCA_003222395.1) | 0.407 | 0.0261 |
|  | OLB13_sp001567485(GB_GCA_001567485.1) | 0.405 | 0.01 |
|  | GWC2-73-18_sp001794945(GB_GCA_001794945.1) | 0.405 | 0.0137 |
|  | *Luteitalea pratensis*(RS_GCF_001618865.1) | 0.405 | 0.0075 |
|  | *Paraclostridium benzoelyticum*(RS_GCF_001006285.1) | 0.403 | 0.0074 |
|  | *Methylobacter luteus*(RS_GCF_000427625.1) | 0.403 | 0.0274 |
|  | *Pirellula staleyi*(RS_GCF_000025185.1) | 0.402 | 0.0155 |
|  | RBG-16-71-46_sp001780165(GB_GCA_001780165.1) | 0.401 | 0.0272 |
|  | *W-Chloroflexi-9*_sp002840675(GB_GCA_002840675.1) | 0.4 | 0.0304 |
|  | *Kouleothrix aurantiaca*(GB_GCA_001399705.1) | 0.396 | 0.0308 |
|  | UBA2475_sp002319075(RS_GCF_002319075.1) | 0.396 | 0.0143 |
|  | *Nocardioides*_sp000620645(RS_GCF_000620645.1) | 0.395 | 0.0089 |
|  | *Chthoniobacter flavus*(RS_GCF_000173075.1) | 0.393 | 0.0092 |
|  | *Palsa-739*_sp003139545(GB_GCA_003139545.1) | 0.391 | 0.0191 |
|  | *Brevundimonas*_sp001427825(RS_GCF_001427825.1) | 0.39 | 0.0188 |
|  | Gp6-AA56_sp003222395(GB_GCA_003222395.1) | 0.389 | 0.0298 |
|  | *Kouleothrix aurantiaca*(GB_GCA_001399705.1) | 0.389 | 0.0153 |
|  | PALSA-1355_sp003153375(GB_GCA_003153375.1) | 0.388 | 0.0181 |
|  | QHXM01_sp003222945(GB_GCA_003222945.1) | 0.387 | 0.0114 |
|  | Bin18_sp002238415(GB_GCA_002238415.1) | 0.386 | 0.0203 |
|  | *Micromonospora lupini*(RS_GCF_000297395.2) | 0.386 | 0.0299 |
|  | Gp6-AA56_sp003222395(GB_GCA_003222395.1) | 0.385 | 0.0173 |
|  | Gp18-AA60_sp003225335(GB_GCA_003225335.1) | 0.384 | 0.0189 |
|  | Gp6-AA56_sp003222395(GB_GCA_003222395.1) | 0.384 | 0.0174 |
|  | *Phyllobacterium brassicacearum*(RS_GCF_003010955.1) | 0.383 | 0.0158 |
|  | UBA2421_sp002343075(GB_GCA_002343075.1) | 0.382 | 0.0238 |
|  | 2011-GWC2-44-17_sp001029695(GB_GCA_001029695.1) | 0.382 | 0.0257 |
|  | XYD1-FULL-53-11_sp001770165(GB_GCA_001770165.1) | 0.382 | 0.023 |
|  | Gp6-AA56_sp003222395(GB_GCA_003222395.1) | 0.382 | 0.022 |
|  | *Opitutus*_sp003054705(RS_GCF_003054705.1) | 0.382 | 0.0209 |
|  | 67-14_sp001897355(GB_GCA_001897355.1) | 0.38 | 0.0166 |
|  | *Pedobacter nyackensis*(RS_GCF_900176505.1) | 0.379 | 0.03 |
|  | *Paenibacillus T cellulosilyticus*(RS_GCF_003182255.1) | 0.378 | 0.0051 |
|  | *Mesorhizobium metallidurans*(RS_GCF_000350085.1) | 0.375 | 0.0252 |
|  | *Chryseolinea serpens*(RS_GCF_900129725.1) | 0.374 | 0.0189 |
|  | OLB17_sp001464455(GB_GCA_001464455.1) | 0.373 | 0.0285 |
|  | *Niastella vici*(RS_GCF_002077945.1) | 0.37 | 0.0194 |
|  | Gp6-AA56_sp003222395(GB_GCA_003222395.1) | 0.37 | 0.0243 |
|  | *Chryseolinea serpens*(RS_GCF_900129725.1) | 0.369 | 0.0235 |
|  | *Chthoniobacter flavus*(RS_GCF_000173075.1) | 0.366 | 0.0107 |
|  | AV2_sp003218935(GB_GCA_003218935.1) | 0.365 | 0.0309 |
|  | *Kouleothrix aurantiaca*(GB_GCA_001399705.1) | 0.365 | 0.0272 |
|  | *Pirellula staleyi*(RS_GCF_000025185.1) | 0.365 | 0.0293 |
|  | AV55_sp003219415(GB_GCA_003219415.1) | 0.364 | 0.0316 |
|  | *Turicibacter sanguinis*(RS_GCF_000178255.1) | 0.364 | 0.0485 |
|  | *Microvirga ossetica*(RS_GCF_002741015.1) | 0.363 | 0.0299 |
|  | HRBIN33_sp002923375(GB_GCA_002923375.1) | 0.362 | 0.0323 |
|  | UBA11741_sp002427845(GB_GCA_002427845.1) | 0.36 | 7.00E-04 |
|  | QHBO01_sp003243965(GB_GCA_003243965.1) | 0.359 | 0.0312 |
|  | *Steroidobacter denitrificans*(RS_GCF_001579945.1) | 0.358 | 0.0328 |
|  | *Arenimonas composti*(RS_GCF_000426365.1) | 0.357 | 0.0189 |
|  | *Dyadobacter beijingensis*(RS_GCF_000382205.1) | 0.357 | 0.0459 |
|  | *Singulisphaera_*sp900129635(RS_GCF_900129635.1) | 0.355 | 0.0287 |
|  | Ga0077555_sp001464855(GB_GCA_001464855.1) | 0.355 | 0.0328 |
|  | *Luteitalea pratensis*(RS_GCF_001618865.1) | 0.355 | 0.035 |
|  | UBA854_sp002295885(GB_GCA_002295885.1) | 0.355 | 0.0492 |
|  | *Blastococcus*_sp900188025(RS_GCF_900188025.1) | 0.354 | 0.0267 |
|  | Fen-1137_sp003142855(GB_GCA_003142855.1) | 0.354 | 0.0405 |
|  | Gp7-AA10_sp003223695(GB_GCA_003223695.1) | 0.354 | 0.0347 |
|  | *Devosia epidermidihirudinis*(RS_GCF_000971295.1) | 0.353 | 0.0288 |
|  | PMNU01_sp002952755(RS_GCF_002952755.1) | 0.352 | 0.0478 |
|  | QHVH01_sp003222245(GB_GCA_003222245.1) | 0.351 | 0.0326 |
|  | Gp6-AA56_sp003222395(GB_GCA_003222395.1) | 0.35 | 0.0283 |
|  | UBA2421_sp002343075(GB_GCA_002343075.1) | 0.348 | 0.0465 |
|  | *Conexibacter*_A_sp000688095(RS_GCF_000688095.1) | 0.347 | 0.0417 |
|  | *Palsa*-1315_sp002737345(GB_GCA_002737345.1) | 0.346 | 0.0408 |
|  | *Pseudoxanthomonas*_A_sp900104085(RS_GCF_900104085.1) | 0.344 | 0.041 |
|  | UBA2421_sp002343075(GB_GCA_002343075.1) | 0.344 | 0.0301 |
|  | AR5_sp003220265(GB_GCA_003220265.1) | 0.344 | 0.0406 |
|  | QHWT01_sp003222675(GB_GCA_003222675.1) | 0.342 | 0.0306 |
|  | *Chthoniobacter flavus*(RS_GCF_000173075.1) | 0.341 | 0.0096 |
|  | JOSHI-001_sp002198735(RS_GCF_002198735.1) | 0.341 | 0.0445 |
|  | 2-12-FULL-60-19_sp001798595(GB_GCA_001798595.1) | 0.34 | 0.0462 |
|  | *Sphingopyxis_*sp900108295(RS_GCF_900108295.1) | 0.339 | 0.0297 |
|  | *Phenylobacterium_*sp001557235(RS_GCF_001557235.1) | 0.338 | 0.0335 |
|  | QHWT01_sp003222675(GB_GCA_003222675.1) | 0.336 | 0.0498 |
|  | *Microlunatus phosphovorus*(RS_GCF_000270245.1) | 0.335 | 0.0108 |
|  | QHWT01_sp003222675(GB_GCA_003222675.1) | 0.333 | 0.045 |
|  | *Enhygromyxa salina_C*(GB_GCA_000737335.3) | 0.33 | 0.0258 |
|  | *Mucilaginibacter pineti*(RS_GCF_900101875.1) | 0.329 | 0.0285 |
|  | *Rhizobacter_*sp000799305(RS_GCF_000799305.1) | 0.329 | 0.0472 |
|  | *Ohtaekwangia koreensis*(RS_GCF_900167975.1) | 0.328 | 0.03 |
|  | *Kouleothrix aurantiaca*(GB_GCA_001399705.1) | 0.322 | 0.0474 |
|  | QHVH01_sp003222245(GB_GCA_003222245.1) | 0.318 | 0.0132 |
|  | UBA4722_sp002404295(GB_GCA_002404295.1) | 0.302 | 0.0306 |
| **Cercozoa** | *Nudifila producta* | 0.388 | 0.0115 |
|  | *Euglypha rotunda* | 0.386 | 0.0112 |
|  | *Spongomonas minima* | 0.363 | 0.0081 |
|  | *Paracercomonas compacta* | 0.352 | 0.0216 |
|  | *Nucleocercomonas praelonga* | 0.343 | 0.0309 |
|  | *Sorosphaera veronicae* | 0.338 | 0.0224 |
|  | *Neocercomonas jutlandica* | 0.33 | 0.0473 |
|  | *Trachelocorythion pulchellum* | 0.328 | 0.0376 |
|  | *Sandona aporians* | 0.325 | 0.0304 |
|  | *Paracercomonas virgaria* | 0.306 | 0.0172 |
|  | *Tremula longifila* | 0.28 | 0.0259 |
